# Effectiveness of Remission Induction Strategies for Early Rheumatoid Arthritis: a Systematic Literature Review

**DOI:** 10.1007/s11926-019-0821-1

**Published:** 2019-04-23

**Authors:** M. M. A. Verhoeven, P. M. J. Welsing, J. W. J. Bijlsma, J. M. van Laar, F. P. J. G. Lafeber, J. Tekstra, J. W. G. Jacobs

**Affiliations:** Department of Rheumatology & Clinical Immunology, University Medical Center Utrecht, Utrecht University, G02.228, P.O. Box 85500, 3508GA Utrecht, The Netherlands

**Keywords:** Early rheumatoid arthritis, Induction therapy, Standard care, bDMARDs, csDMARDs, GCs

## Abstract

**Purpose of Review:**

To review the effectiveness of remission induction strategies compared to single csDMARD-initiating strategies according to current guidelines in early RA.

**Recent Findings:**

Twenty-nine studies, heterogeneous on, e.g., specific treatment strategy and remission outcome used, were identified. Using DAS28-remission over 12 months, 13 (76%) of 17 remission induction strategies showed significantly more patients achieving remission. Pooled relative “risk” was 1.73 [95%CI 1.59–1.88] for bDMARD-based remission induction strategies and 1.20 [95%CI 1.03–1.40] for combination csDMARD-based remission induction strategies compared to single csDMARD-initiating strategies. When additional glucocorticoid “bridging therapy” was used in single csDMARD-initiating strategies, the higher proportion patients achieving remission in remission induction strategies was no longer statistically significant (pooled RR 1.06 [95%CI 0.83–1.35]). For other remission outcomes, results were in line with above.

**Summary:**

Remission induction strategies are more effective in achieving remission compared to single csDMARD-initiating strategies, possibly more so in bDMARD-based induction strategies. However, compared to single csDMARD-initiating strategies with glucocorticoids, induction strategies may not be more effective.

**Electronic supplementary material:**

The online version of this article (10.1007/s11926-019-0821-1) contains supplementary material, which is available to authorized users.

## Introduction

In rheumatoid arthritis (RA), early initiation of disease-modifying anti-rheumatic drug (DMARD) treatment, preferably within the “window of opportunity,” is thought to optimally prevent joint damage, improving long-term outcome and quality of life [1, 2].

Accordingly, current international guidelines advice to start treatment in early RA as soon as possible after diagnosis. Initial therapy is started with a conventional synthetic (cs)DMARD, most frequently methotrexate (MTX), in a “tight-controlled” manner, aiming for low disease activity or, preferably, remission [1, 3].

Initial MTX therapy is sometimes combined with short-term use of moderate-high dose glucocorticoids (GCs), which are then tapered as soon as possible: GC bridging therapy. The treatment strategy has to be intensified if the treatment target is not achieved within 6 months [1, 3]. This next step is often to add a biological (b) or targeted small molecule (ts) DMARD [4••, 5].

Previous research shows that approximately 30–50% of early RA patients need additional b/tsDMARD therapy [6].

Patients who initiate a more intensive DMARD strategy as first-line treatment than that according to current guidelines as described above have sometimes shown superior effectiveness outcomes, and achieve remission more often and earlier, sometimes also including sustained remission (SR) and even sustained drug-free remission (sDFR), which may thus become achievable treatment targets [4••, 7].

Achieving remission earlier has been found to be related to improved long-term outcomes [7].

Furthermore, SR and sDFR may become future treatment targets for early RA within the window of opportunity. This may lead to a paradigm shift towards the above described so called remission induction strategies.

For this reason, it would be interesting to investigate the effectiveness of initiating in early RA more intensive treatment strategies, compared to single csDMARD-initiating strategies according to current guidelines; these more intensive strategies herein are designated remission induction strategies.

The aim of the study is to provide a systematic summary of these remission induction strategies and their effectiveness.

## Methods

### Systematic Literature Search and Study Selection

A systematic review of the literature was performed according to current standards and reported according to the Preferred Items for Systematic Reviews and Meta-analyses (PRISMA) statement protocol [8]. In October 2018, we performed a literature search in Medline and Embase. The search combined terms relating to early RA, terms for cs-, b-, and tsDMARD and remission, and publications limited to the last 5 years and English language. More details about the research question and search terms can be found in the Supplementary file.

We defined more intensive, remission induction strategies as initiating treatment with a bDMARD or a tsDMARD, both with and without a csDMARD, or initiating a csDMARD with moderately or high-dosed GCs, with delayed tapering (not “bridging therapy”) or starting ≥ 2 csDMARDs.

The single csDMARD-initiating strategy was defined as starting treatment with a single csDMARD, with or without GCs as bridging therapy, according to the current guidelines.

All titles and abstracts were screened by MMAV. If the reviewer was unsure about in-/excluding an abstract, it was discussed with one other co-author (PMJW) and one co-investigator (MdH) to reach consensus, and in case of remaining doubt based on title/abstract, the publication was included for full text evaluation. Full text screening was performed using the same strategy.

The following selection criteria were used: (1) human studies, (2) (very, DMARD-naive) early RA patients, (3) remission induction strategy arm (according to definition of remission induction strategy, see above), (4) single csDMARD-initiating strategy arm (according to definition of single csDMARD-initiating strategy, see above) and, (5) results presented regarding the comparison of a remission induction strategy and a single csDMARD-initiating strategy on an outcome of remission.

Remission was defined as remission according to a validated disease activity index or the Boolean definition [1].

Randomized controlled trials (RCTs) as well as cohort studies with appropriate correction for multiple confounders were selected. Long-term extension studies of trials satisfying the above criteria were also selected to investigate long-term effects of remission induction strategies on, e.g., radiographic progression.

### Data Extraction and Outcome Assessment

The following data of studies was extracted: publication year, study design, patients’ baseline characteristics (age, gender, rheumatoid factor (RF) status, Health Assessment Questionnaire (HAQ), symptom duration, Disease Activity Score assessing 28 joints (DAS28), a description of the single csDMARD-initiating strategy and the remission induction strategy, the number of patients per arm, a description of the remission outcome, the number of patients achieving remission per arm, a description of missing data, and other remarks deemed necessary. In case of a study evaluating long-term outcomes of a remission induction strategy, we extracted additional information (if available) for the follow-up duration, outcome for disease activity, medication use and radiographic progression.

A quality assessment of all selected publications was performed using “The Cochrane Collaboration’s tool for assessing risk of bias” [9]. Information about random sequence generation, allocation concealment, blinding of participants and personnel, blinding of outcome assessment, incomplete outcome assessment, and selective reporting was evaluated.

### Statistics

Relative risks (RR) for achieving remission with 95% confidence intervals (CI) per study were calculated, separate for each remission outcome definition and graphically displayed in forest plots. When appropriate, results were pooled using a random-effect model according to the Mantel-Haenszel method. To explore the effects of specific remission induction strategy used (e.g., use of a b/tsDMARD, the use of GC bridging therapy in the single csDMARD-initiating strategy) and the effect of symptom duration at start of therapy (within the window of opportunity, arbitrarily defined as symptom duration ≤ 3 months, versus outside the window of opportunity, arbitrarily defined as symptom duration > 3 months) [2], group analyses were performed.

Outcomes of studies evaluating the longer term effectiveness of remission induction strategies were only summarized descriptively.

All analyses were performed in Review manager version 5.3 [10].

## Results

After screening, 23 articles and 6 conference abstracts were included, involving 6319 patients treated according to a remission induction strategy and 4647 according to a single csDMARD-initiating strategy (see flowcharts in Supplementary figure 1). Four specific groups were defined based on characteristics of the drug regime and study duration, and comparisons made: (1) b/tsDMARD-based remission induction strategy versus single csDMARD-initiating strategy without GC bridging, (2) combination csDMARD-based remission induction strategy versus single csDMARD-initiating strategy without GC bridging, (3) remission induction strategy (either combination csDMARD-based strategy or bDMARD-based strategy) versus single csDMARD-initiating strategy with GC bridging, and (4) studies evaluating long-term effects of remission induction strategies (follow-up > 4 years). An overview of patient and study characteristics of the included studies is shown in Table 1.

**Table 1:** Baseline patient and disease characteristic of included studies

First author, publication year, reference	Design	Mean age in years (SD)	Female (%)	RF+ (%)	Mean HAQ score (SD)	Mean symptom duration in weeks (SD)	Mean DAS28 (SD)	Single csDMARD-initiating strategy	N in single csDMARD-initiating strategy	Remission induction strategy	N in remission induction strategy	Time of assessments in years	Treatment characteristics (both arms)
b/tsDMARD-based remission induction strategy versus single csDMARD-initiating strategy without GC bridging													
Atsumi 2016 [11]	RCT	49 (11)	81	95	1.1 (0.7)	16 (11)	5.5 (1.2)	MTX+PBO	157	CZP+MTX	159	1	T2T
Bijlsma 2016 [4]	RCT	54 (^§^)	67	72	1.2 (0.6)	4 (^§^)	5.2 (1.1)	MTX+PBO	108	TCZ+MTX TCZ+PBO	106 103	0.5	T2T
Burmester 2016 [12••]	RCT	50 (13)	78	89	1.6 (0.7)	26 (26)	6.7 (1.0)	MTX+PBO	289	TCZ+MTX TCZ+MTX (reduced dose) TCZ+PBO	291 290 292	1	T2T
Dougados 2014 [13]	RCT	52 (14)	72	§	§	34 (22)	6.5 (1.0)	MTX	178	ETN+MTX	213	1	T2T
Emery 2017 [14••]	RCT	51 (14)	77	97	1.6 (0.6)	12 (17)	6.7 (0.9)	MTX+PBO	213	CZP+MTX	655	1	T2T
Horslev-Petersen 2014 [15]	RCT	55 (^§^)	66	72	1.1 (^§^)	12 (^§^)	5.6^§^	MTX+PBO	91	ADA+MTX	89	1	T2T
Keystone 2017 [16]	RCT	§	§	§		§	§	MTX+PBO	210	BARI+MTX BARI	215 159	1	T2T
Keystone 2017a [17]	RCT	52 (14)	84	73	1.5 (0.7)	39 (44)	6.3 (0.9)	MTX+PBO	257	ADA+MTX	268	0.5	T2T
Kirchgsner 2018 [18]	RCT	48 (12)	§	§	§	§	§	PBO	15	INF	15	1	No adjustments INF until 22w
Nam 2014b [19]	RCT	48 (13)	76	55	1 (0.4)	28 (^§^)	4.1 (1.1)	MTX+PBO	55	ETN+MTX	55	1	T2T
Smolen 2015 [20]	RCT	49 (13)	78	97	1.7 (0.7)	26 (29)	6.3 (1.0)	MTX+PBO	209	ADA+MTX	210	1	T2T
Stamm 2018 [21]	RCT	53 (14)	73	35	0.9 (0.7)	10 (2)	4.9 (1.4)	MTX+PBO	36	INF+MTX	38	1	Step up MTX
Takeuchi 2014 [22]	RCT	54 (13)	81	84	1.2 (0.8)	16 (21)	6.6 (1.0)	MTX+PBO	163	ADA+MTX	171	0.5	T2T
Combination csDMARD-based remission induction strategy versus single csDMARD-initiating strategy without GC bridging													
Brunekreef 2017 [23]	Cohort	59 (14)	62	65	§	§	§	MTX	297	MTX+HCQ+GCim	156	1	T2T IM 80-120 mg
Ma 2014 [24]	RCT	54 (^§^)	68	87	1.6 (^§^)	§	5.8 (1.3)	MTX	87	CSA+MTX+GC	90	2	T2T Bridg. 34w
Rannio 2017 [25]	Cohort	57 (16)	67	71	0.9 (^§^)	24 (^§^)	4.2 (1.4)	MTX (+GC)	453	MTX+SSZ+HCQ	158	1	T2T
Steunebrink 2016 [26]	2 cohorts	59 (13)	62	54	1.1 (^§^)	§	4.7 (1.1)	MTX+PBO	128	MTX+HCQ	128	1	T2T
Remission induction strategy (either combination csDMARD-based strategy or bDMARD-based strategy) versus single csDMARD-initiating strategy with GC bridging													
Akdemir 2018 [27]	2 RCT	54 (14)	69	63	1.5 (0.7)	20 (^§^)	4.3 (0.8)**	MTX+GC	175	MTX+SSZ+GC	133	1	T2T Bridg. 34w
De Jong 2014 [28]	RCT	54 (14)	68	71	1 (0.7)	24 (13)	3.4 (1.0)**	MTX+GC	97	MTX+SSZ+HCQ+GCim MTX+SSZ+HCQ+GC	91 93	1	T2T IM 80-120 mg or bridg. 10w
Nam 2014a* [29]	RCT	53 (13)	69	55	1.4 (0.5)	5 (^§^)	3.8 (1.0)**	MTX+GCiv	57	MTX+INF	55	0.5	T2T IV 250 mg
Stouten 2017 [30]	RCT	§	§	§	§	§	§	MTX+GC	98	MTX+SSZ+GC MTX+LEF+GC	98 93	1	T2T Bridg. 34w
Ter Wee 2015 [31]	RCT	52 (13)	69	59	1.4 (0.7)	24 (20)	5.4 (1.2)	MTX+GC	81	MTX+SSZ+GC	81	1	T2T Bridg. 34w
Verschueren 2017 [32]	Trial	52 (13)	71	58	1 (0.7)	3 (4)	4.7 (1.4)	MTX+GC MTX	141 172	MTX+SSZ+GC MTX+LEF+GC	98 90	1	T2T Bridg. 34w
Studies evaluating long-term effects of remission induction strategies (follow-up > 4 years)													
Bergsma 2017 [33]	RCT	55 (11)	55	61	1.3 (0.7)	21(^§^)	4.2 (0.9)	MTX+PBO	247	MTX+SSZ+GC	261	10	T2T Bridg. 34w
Emery 2016 [34]	RCT	§	§	§	§	§	§	MTX+PBO	160	GOL GOL(reduced dose)+MTX GOL+MTX	159 159 159	5	T2T
Keystone 2014 [35]	RCT	§	§	§	1.3 (0.7)	§	5.6 (1.7)	MTX+PBO	164	ADA+MTX ADA+PBO	196 166	10	T2T
Konijn 2017 [36]	RCT	57 (13)	67	§	§	§	§	MTX+GC	81	MTX+SSZ+GC	81	4	T2T Bridg. 34w
Markusse 2016 [37]	RCT	54 (14)	69	65	1.4 (0.7)	24(^§^)	4.4 (0.9)**	MTX MTX+GC	126 121	MTX+SSZ+GC INF+MTX	133 128	10	T2T Bridg. 28w
Verhoeven 2018 [38]	RCT	§	§	§	§	§	§	MTX+PBO	72	TCZ+MTX TCZ+PBO	75 79	5	T2T

^§^(spread of) variable not available; *bDMARD-based remission induction strategy; **DAS44 (assessing 44 joints); reduced dose is 0.5 of the normal dose; *RF*, rheumatoid factor; *HAQ*, health assessment questionnaire; *DAS28*, disease activity score assessing 28 joints; *RCT*, randomized controlled trial; *bridg*, bridging therapy; *im*, intramuscular; *iv*, intravenous; *Ada*, adalimumab; *BARI*, baracitinib; *CSA*, ciclosporine; *CZP*, certolizumab pegol; *ETN*, etanercept; *GC*, glucocorticoid; *GOL*, golimumab, *HCQ*, hydroxychloroquine; *INF*, infliximab; *Lef*, lefunomide; *MTX*, methotrexate; *PBO*, placebo; *SSZ*, sulfasalazine; *TCZ*, tocilizumab; *T2T*, treat-to-target treatment strategy including step-up and step-down

Several of the 29 studies used more than 1 remission definition; in all, 46 remission definitions were used, range 1–4 per study. Most studies used at least a definition of remission where remission had to be present ≥ 1 visit within 6 to 12 months follow-up and according to one of our remission outcome definitions; we will describe the results based on these outcomes (Table 1). Seventeen studies defined remission as DAS28 < 2.6, 12 studies used the Boolean remission definition, 7 studies used CDAI ≤ 2.8, and 10 studies used SDAI ≤ 3.3; results are described separately below. Overall, for 32 of the 46 remission definitions (70%), a statistically significant effect in favor of remission induction strategy was found.

### DAS28-Based Remission

**Fig. 1:**
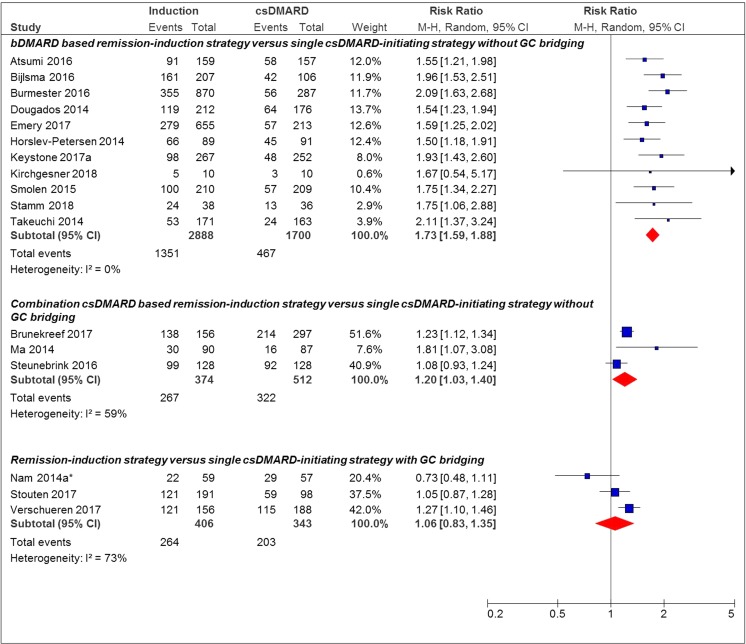
Forest plot of DAS28 remission outcome in individual studies comparing remission induction strategies with single csDMARD-initiating strategies. DAS28 remission, DAS28 < 2.6; induction, remission induction strategy arm; csDMARD, single csDMARD-initiating strategy arm; M-H, Mantel-Haenszel; Random, random effect; *bDMARD-based remission induction strategy. 95% CI, 95% confidence interval

When DAS28 was used for remission definition, 13/17 (76%) studies showed a statistically significant effect in favor of the remission induction strategy (Fig. 1). The pooled RR of achieving remission for strategies using a bDMARD in the remission induction strategy compared to the single csDMARD-initiating strategy without GC bridging was 1.73 [95%CI 1.59–1.88] versus 1.20 [95%CI 1.03–1.40] for studies which used a combination csDMARD-based remission induction strategy compared to the single csDMARD-initiating strategy without GC bridging. For studies using GC bridging in the single csDMARD-initiating strategy, no statistically significant additional effect for the remission induction strategy was found (pooled RR 1.06 [95% CI 0.83–1.35]). One of them used a bDMARD in the remission induction strategy arm. [29] One cohort study only provided an OR for achieving remission in patients treated with a remission induction strategy compared to a single csDMARD-initiating strategy, with or without additional GC use (without sufficient information to calculate an RR). Results were in favor of the remission induction strategy (OR 1.82 [95%CI 1.01–3.29]) [25].

### Boolean-Based Remission

**Fig. 2:**
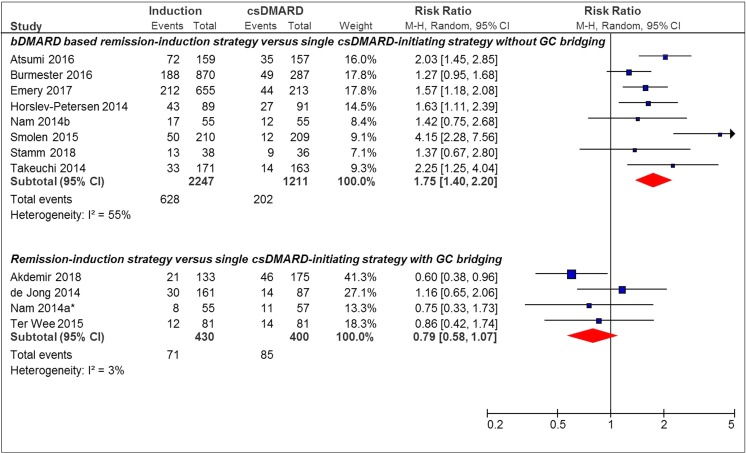
Forest plot of Boolean remission outcome in individual studies comparing remission induction strategies with single csDMARD-initiating strategies. Boolean remission—tender joint count ≤ 1, swollen joint count ≤ 1, CRP ≤ 1 mg/dL, patient global assessment ≤ 1 (on a 0–10 scale); induction, remission induction strategy arm; csDMARD, single csDMARD-initiating strategy arm; M-H, Mantel-Haenszel; random, random effect; *bDMARD-based remission induction strategy. 95% CI, 95% confidence interval

For Boolean remission, 5/12 (42%) studies showed a statistically significant effect in favor of the remission induction strategy. The pooled RR of achieving Boolean remission for the bDMARD-based remission induction strategy compared to the single csDMARD-initiating strategy without GC bridging was 1.75 [95%CI 1.40–2.20], versus 0.79 [95% CI 0.58–1.07] for the remission induction strategy (1/5 bDMARD use in the remission induction strategy) [29] compared to the single csDMARD-initiating strategy with GC bridging (Fig. 2).

### CDAI-Based Remission

**Fig. 3:**
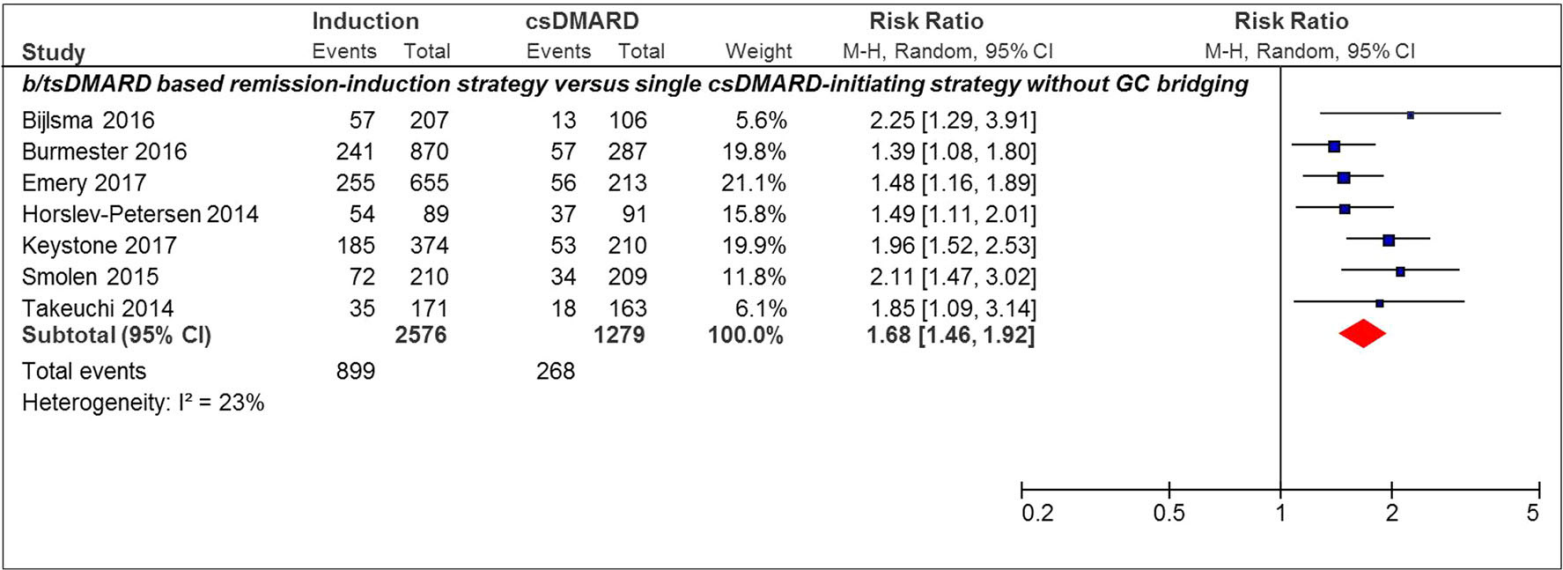
Forest plot of CDAI remission outcome in individual studies comparing remission induction strategies with single csDMARD-initiating strategies. CDAI remission, CDAI ≤ 2.8; induction, remission induction strategy arm; csDMARD, single csDMARD-initiating strategy arm; M-H, Mantel-Haenszel; random, random effect. 95% CI, 95% confidence interval

Only studies with b/tsDMARD use in the remission induction strategy versus single csDMARD-initiating strategy without GC bridging were included in the analysis for CDAI remission. All studies (7/7, 100%) showed a statistical significant effect in favor of the remission induction strategy arm. The pooled RR of achieving CDAI remission was 1.68 [95%CI 1.46–1.92] (Fig. 3).

### SDAI-Based Remission

**Fig. 4:**
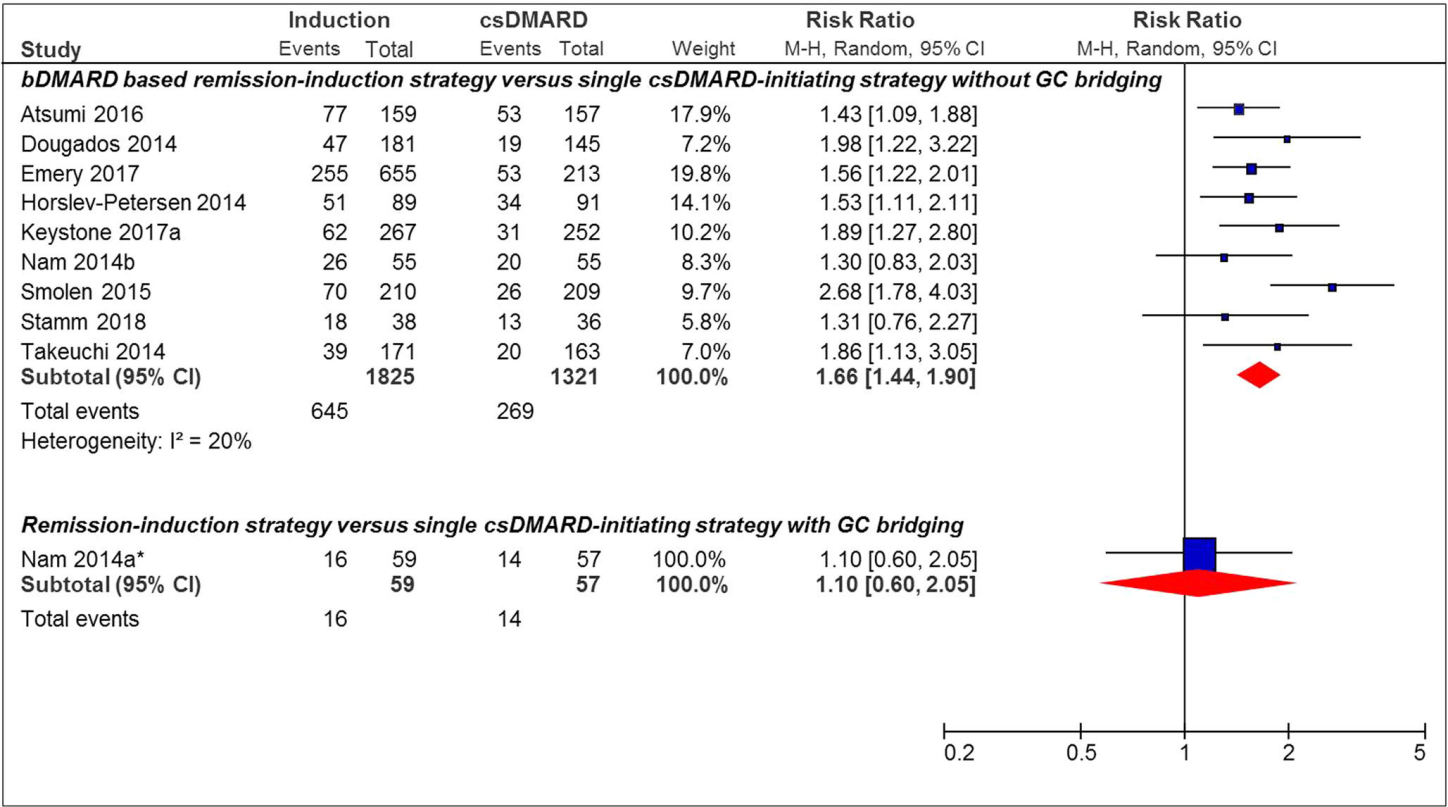
Forest plot of SDAI remission outcome in individual studies comparing remission induction strategies with single csDMARD-initiating strategies. SDAI remission, SDAI ≤ 3.3; induction, remission induction strategy arm; csDMARD, single csDMARD-initiating strategy arm; M-H, Mantel-Haenszel; random, random effect; *bDMARD-based remission induction strategy

Nine studies with bDMARD use in the remission induction strategy arm versus single csDMARD-initiating strategy without GC bridging, and one study using a bDMARD-based remission induction strategy versus a single csDMARD-initiating strategy with GC bridging were included in the analysis for SDAI remission [29]. A significant effect in favor of the remission induction strategy was found in 7/10 (70%) studies (Fig. 4). The pooled RR of achieving SDAI remission was 1.66 [95%CI 1.44–1.90] for bDMARD use in the remission induction strategy arm versus the single csDMARD-initiating strategy without GC bridging arm. And for the single study where a remission induction strategy was compared to a single csDMARD-initiating strategy with GC bridging, this was 1.10 [95%CI 0.60–2.05].

### Symptom Duration

Regarding symptom duration, six studies started treatment “within the window of opportunity” (symptom duration ≤ 3 months). Another nine studies started treatment “outside the window of opportunity” (symptom duration > 3 months; range 4–10 months). All studies reported the DAS28-based remission outcome, and 11/15 (73%) showed a statistically significant effect in favor of the remission induction strategy. The pooled RR of achieving remission for strategies within the window of opportunity was 1.43 [95%CI 1.15–1.77] versus 1.44 [95%CI 1.12–1.86] for studies outside the window of opportunity. Five studies used a single csDMARD-initiating strategy with GC bridging (i.e., two studies within and three studies outside; Supplementary figure 2).

### Longer Term Effectiveness of Remission Induction Strategies Started in Early RA

We found six studies evaluating the effect of a remission induction strategy versus a single csDMARD-initiating strategy on the long term (4 to 10 years). In four studies, DAS remission was more often achieved in the initial remission induction strategy compared to the single csDMARD-initiating strategy over time [33–35, 37]. In the remission induction strategy arm, Boolean remission, as well as SDAI remission, was less often achieved in one of two studies with no difference in the other, compared to the single csDMARD-initiating strategy arm [34, 36]. No difference was found for CDAI remission, which was reported in only one study [34]. One study reported data about SR, which was achieved in almost all patients over time, without differences between the different strategy arms [38]. However, using (s)DFR as outcome, differences were shown in favor of the remission induction strategies [37, 38]. No differences were found for radiographic progression over time between the different strategies [33–35, 37]. Details of these studies can be found in Table 1.

### Risk of Bias Assessment

The risk of bias of the included studies was overall low. In general, 26/29 studies were RCTs, the remaining 3 were cohort studies. An overview of the risk of bias assessment is shown in Supplementary table 1. In the studies evaluating long-term effects of a remission induction strategy, after the initial RCT [33–38], treatment was according to the treating physician and standard care, without detailed information on the initial trial and attrition, prohibiting to fully assess all items of the risk of bias assessment. Further, moderate/high risk of bias was present in the seven studies evaluating short-term effects [23, 25–27, 30–32].

## Discussion

The current meta-analysis shows that a remission induction strategy is more effective compared to a single csDMARD-initiating strategy, possible specifically for bDMARD-based remission induction strategies. However, this superior effect over single csDMARD-initiating strategy is limited and is not statistically significant, if patients are treated initially also with GCs, short-term as “bridging therapy.” Longer term follow-up studies showed conflicting results, but a more favorable outcome with regard to (s)DFR for the remission induction strategy may be present.

No overall pooled effect estimate was given as studies were highly heterogeneous in study design regarding, e.g., specific drug regimen and remission outcome used. We therefore defined groups of more homogeneous studies based on specific remission outcomes and characteristics of drug regimen. Results within these groups show that heterogeneity is typically low, and therefore we pooled the effect estimates. However, in some of these groups, heterogeneity was moderate, based on differences in study design, medical treatment, risk of bias and/or patient characteristics (I^2^ > 50%, see Figs. 1 and 2).

One surprising finding was that the added value of a remission induction strategy was found to be limited and non-statistically significant when compared to a single csDMARD-initiating strategy with GC bridging therapy. This may suggest that the current early start of therapy, including a treat to target approach with swift step-up treatment adjustments, achieves already very good results when the initial delay in treatment effect is covered by the bridging therapy.

Contrary to expectation, similar beneficial outcomes for patients treated within the window of opportunity were found when compared with those for patients treated outside the window of opportunity. However, only a limited number of studies reported data on symptom duration which is notoriously difficult to define, and our study was not specifically designed to test the window of opportunity hypothesis. Outside of our study, in some papers, a difference in effectiveness of treatment has been shown in favor of patients treated within the window of opportunity [2, 39].

In general, long-term effectiveness outcomes were not different between a remission induction strategy and a single csDMARD-initiating strategy probably due to the widely applied treat to target principle [1].

Results of our systematic literature review are in line with an earlier performed systematic literature review, which included only remission induction strategies using a b/tsDMARD in the experimental arm [40]. We, uniquely include also combination csDMARD-based remission induction strategy arms, providing results applicable also for countries with limited availability of bDMARDs. Besides, we evaluated several established remission definitions according to validated disease activity indices and the Boolean definition [1].

No data on radiographic progression was reported, because of the limited study duration of most included studies; even over 2 years, radiographic progression is absent or modest at most in treat to target studies in early RA [41, 42]. Only some of the long-term extension studies reported on radiographic progression, but did not show any statistically significant differences.

The majority of all included studies, i.e., 20/29 (69%), were RCTs with no to moderate risk of bias. The longer term follow-up studies were follow ups of RCTs, in which the effectiveness was maintained, indicating the quality of keeping to the treat to target principle.

## Conclusions

Remission induction strategies initiated in early RA patients are more effective in achieving remission compared to single csDMARD-initiating strategies. However, their benefit compared to that of a single csDMARD-initiating therapy strategy with GC bridging seems to be limited.

## Electronic supplementary material

Supplementary figure 1 Flowchart of included studies (JPG 59 kb)

Supplementary figure 2 Forest plot of DAS remission outcome in each individual study in which patients were treated within the window of opportunity (symptom duration ≤ 3 months) and treated outside the window of opportunity (symptom duration > 3 months). (JPG 148 kb)

Supplementary table 1 Quality assessment of individual studies (DOCX 17 kb)
