# Supplementary figures and images for: Effectiveness of Remission Induction Strategies for Early Rheumatoid Arthritis: a Systematic Literature Review
Source: Curr Rheumatol Rep. 2019 Apr 23;21(6):24. doi: 10.1007/s11926-019-0821-1 (PMC6478774; doi:10.1007/s11926-019-0821-1)

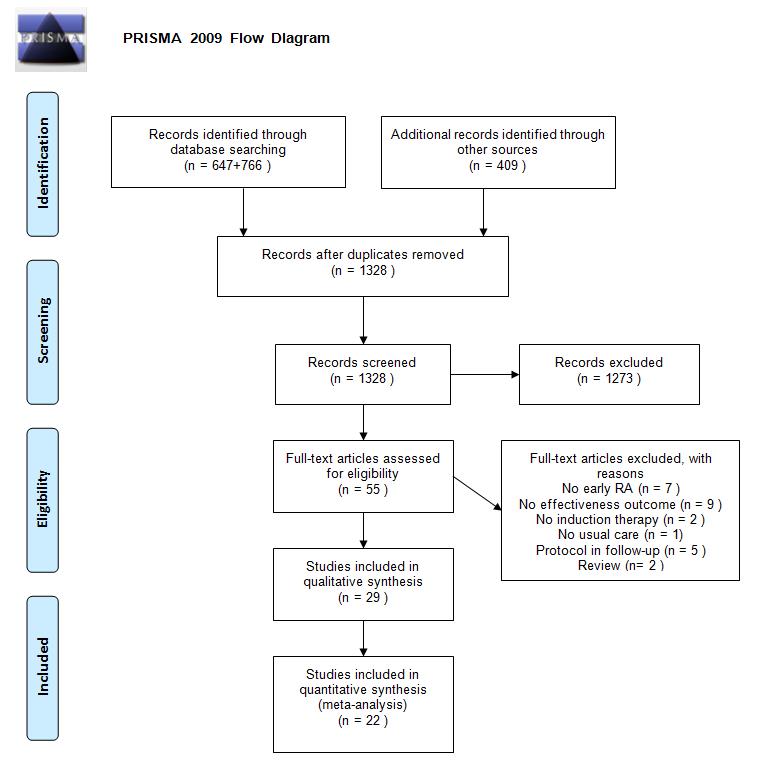

Supplement: Supplementary file 1 — Flowchart of included studies (JPG 59 kb) [file 11926_2019_821_MOESM1_ESM.jpg]

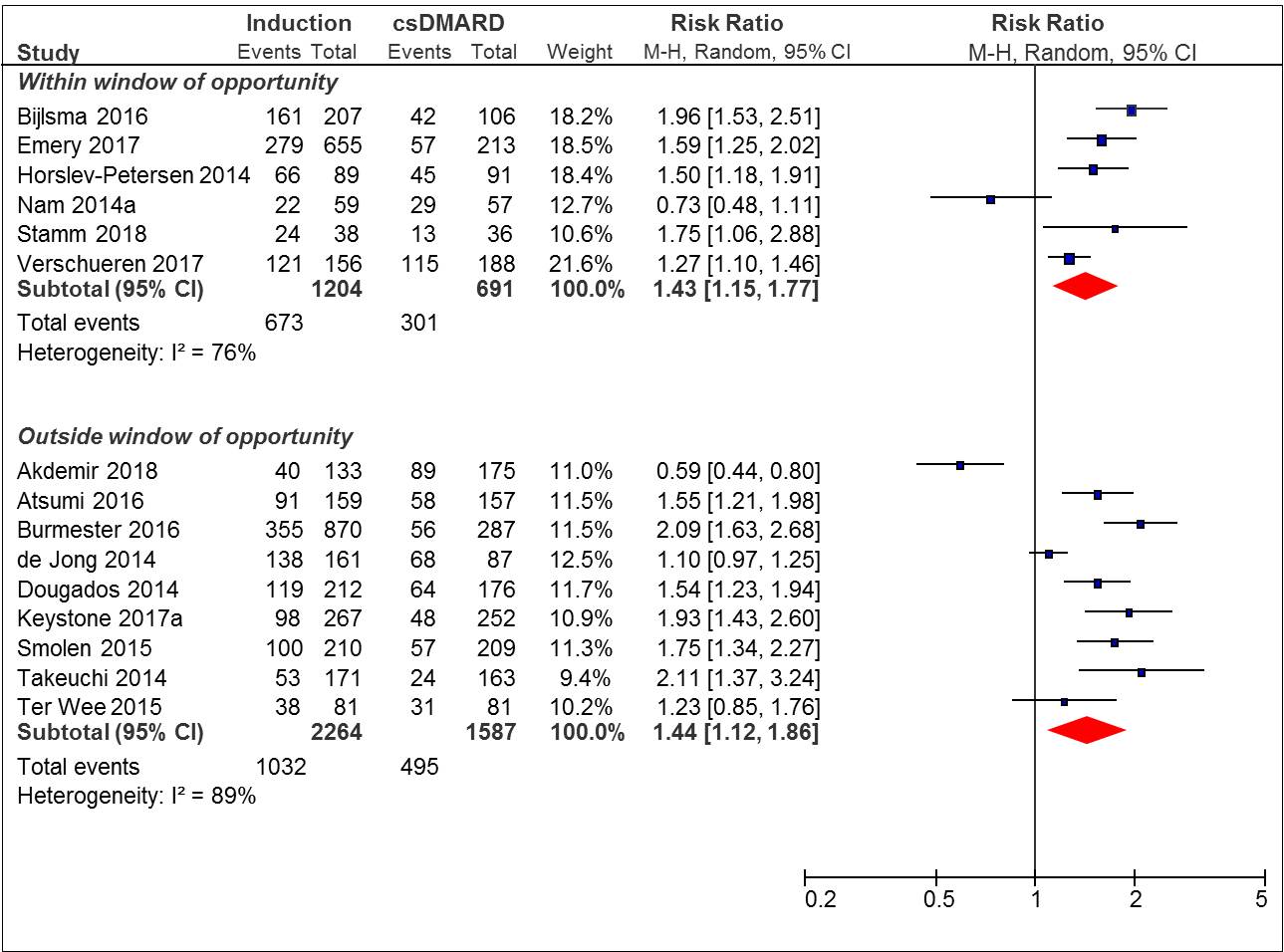

Supplement: Supplementary file 2 — Forest plot of DAS remission outcome in each individual study in which patients were treated within the window of opportunity (symptom duration ≤ 3 months) and treated outside the window of opportunity (symptom duration > 3 months). (JPG 148 kb) [file 11926_2019_821_MOESM2_ESM.jpg]
