# Supplementary material for: Effectiveness of Remission Induction Strategies for Early Rheumatoid Arthritis: a Systematic Literature Review
Source: Curr Rheumatol Rep. 2019 Apr 23;21(6):24. doi: 10.1007/s11926-019-0821-1 (PMC6478774; doi:10.1007/s11926-019-0821-1)
Supplement: Supplementary file 3 — Quality assessment of individual studies (DOCX 17 kb) [file 11926_2019_821_MOESM3_ESM.docx]

| **First author, publication year** | **Sequence generation** | **Allocation concealment** | **Blinding of participants and personnel** | **Blinding of outcome assessment** | **Incomplete outcome assessment** | **Selective reporting** |
| --- | --- | --- | --- | --- | --- | --- |
| Akdemir 2018 | **+** | **+** | **?** | **+** | **?** | **?** |
| Atsumi 2016 | **+** | **+** | **+** | **+** | **+** | **+** |
| Bergsma 2017 | **x** | **x** | **x** | **x** | **+** | **?** |
| Bijlsma 2016 | **+** | **+** | **+** | **+** | **+** | **+** |
| Brunekreef 2017 | **-** | **-** | **?** | **+** | **+** | **?** |
| Burmester 2016 | **+** | **+** | **+** | **+** | **+** | **+** |
| De Jong 2014 | **+** | **+** | **?** | **+** | **+** | **+** |
| Dougados 2014 | **+** | **+** | **+** | **+** | **+** | **+** |
| Emery 2016 | **x** | **x** | **x** | **x** | **+** | **+** |
| Emery 2017 | **+** | **+** | **+** | **+** | **+** | **+** |
| Horslev-Petersen 2014 | **+** | **+** | **+** | **+** | **+** | **+** |
| Keystone 2014 | **x** | **x** | **x** | **x** | **+** | **+** |
| Keystone 2017 | **+** | **+** | **+** | **+** | **+** | **?** |
| Keystone 2017a | **+** | **+** | **+** | **+** | **+** | **?** |
| Kirchgsner 2018 | **+** | **+** | **+** | **+** | **?** | **?** |
| Konijn 2017 | **x** | **x** | **x** | **x** | **+** |  |
| Ma 2014 | **+** | **+** | **+** | **+** | **?** | **?** |
| Markusse 2016 | **x** | **x** | **x** | **x** | **+** | **+** |
| Nam 2014a | **+** | **+** | **+** | **+** | **+** | **+** |
| Nam 2014b | **+** | **+** | **+** | **+** | **+** | **+** |
| Rannio 2017 | **-** | **?** | **?** | **+** | **+** | **?** |
| Smolen 2015 | **+** | **+** | **+** | **+** | **?** | **?** |
| Stamm 2018 | **+** | **+** | **+** | **+** | **+** | **+** |
| Steunebrink 2016 | **?** | **?** | **?** | **+** | **+** | **?** |
| Stouten 2017 | **+** | **+** | **?** | **?** | **+** | **?** |
| Takeuchi 2014 | **+** | **+** | **+** | **+** | **+** | **+** |
| Ter Wee 2015 | **+** | **+** | **-** | **+** | **+** | **+** |
| Verhoeven 2018 | **x** | **x** | **x** | **x** | **+** | **?** |
| Verschueren 2017 | **+** | **+** | **-** | **?** | **+** | **+** |
